# Impact of a Multicomponent Exercise Training Program on Muscle Strength After Bariatric Surgery: A Randomized Controlled Trial

**DOI:** 10.1007/s11695-024-07173-w

**Published:** 2024-03-27

**Authors:** Giorjines Boppre, Florêncio Diniz-Sousa, Lucas Veras, Andrea Bezerra, Vitor Devezas, John Preto, Hugo Santos-Sousa, José Oliveira, Hélder Fonseca

**Affiliations:** 1 Research Centre in Physical Activity, Health, and Leisure (CIAFEL), Faculty of Sport, University of Porto, Rua Dr. Plácido Costa, 91, 4200-450 Porto, Portugal; 2 Laboratory for Integrative and Translational Research in Population Health (ITR), Porto, Portugal; 3 General Surgery Department, São João Medical Center, Porto, Portugal

**Keywords:** Bariatric surgery, Exercise program, Muscle strength

## Abstract

**Purpose:**

This study examined the benefits of an 11-months multicomponent exercise program (MEP) on muscular strength (MS) after bariatric surgery.

**Methods:**

Of the 84 randomized patients, 41 participants from the exercise group (EG) and 20 participants from the control group (CG) were included in the analysis. The EG received supervised MEP for 11 months, starting 1-month post-bariatric surgery (BS) in addition to standard medical care, while the CG received medical care recommendations only. Knee and trunk MS was assessed by isokinetic dynamometry pre-surgery, 1-, 6-, and 12-month post-surgery, while body composition was assessed by dual-energy X-ray absorptiometry**.**

**Results:**

The MEP did not significantly impact absolute MS in the dominant knee and trunk regions at 6- and 12-month post-BS. However, relative MS showed significant improvements. At 6-month post-BS, knee flexion at 60°/s relative to body weight (BW) increased significantly (*p* = 0.047), as did knee extension at 180°/s relative to BW (*p* = 0.009), and knee extension at 60°/s relative to total lean mass (*p*=0.040). At 12-month post-BS, knee flexion at 60°/s relative to BW also significantly improved (*p*=0.038).

**Conclusion:**

While absolute MS was not significantly improved with MEP, this study found significant enhancements in relative MS, particularly in dominant knee flexion post-MEP participation. Further research should explore different exercise intensities and frequencies to optimize postoperative MS recovery post-BS.

**Clinical Trial Registration:**

ClinicalTrials.gov (NCT02843048)

**Graphical Abstract:**

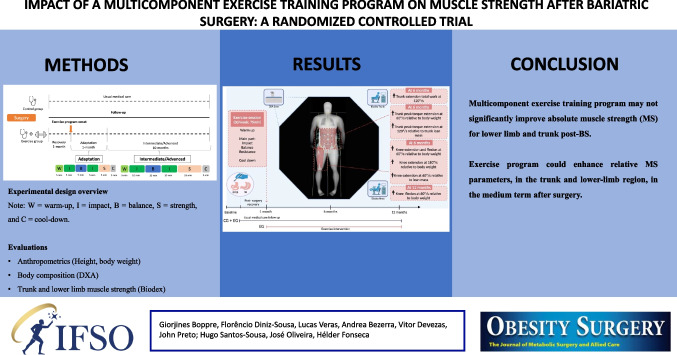

**Supplementary Information:**

The online version contains supplementary material available at 10.1007/s11695-024-07173-w.

## Introduction

Obesity is a severe health and economic burden, including cardiovascular disease, type 2 diabetes, chronic renal disease, and various cancers [1]. Bariatric surgery (BS) has emerged as a vital intervention for treating individuals struggling with severe obesity [2] as it promotes large and sustained weight losses due to the restrictive and malabsorptive nature of the surgery, as well as through its effects on satiety and hunger mechanisms, which lead to a drastic reduction in food consumption [3]. BS induces evident changes in body composition (BC), namely by decreasing fat mass, but can also lead to sustained lean and bone mass losses, particularly in the first months following surgery [4].

Postoperative changes in BC, particularly the lean mass (LM) loss and, consequently, muscle strength (MS), present challenges to the functional capacity and overall well-being of patients who have undergone BS [5–7]. This muscle mass (MM) loss and strength can lead to difficulties performing daily tasks, increased risk of falls, and reduced physical function [8]. Notably, these challenges are interconnected with frequent conditions such as sarcopenia or sarcopenic obesity, where muscle strength plays a crucial role [9]. Sarcopenia, characterized by the progressive loss of muscle mass and function, is a significant concern in postoperative patients [10]. Maintaining or improving MS is therefore crucial for restoring physical function and enhancing the quality of life of post-BS patients [11]. MS is not only essential for daily activities or functional capacity but also serves as a significant indicator of cardiovascular health and mortality risk [8]. Thus, identifying effective strategies to counteract the decline in MS and improve functional outcomes is crucial for individuals who underwent BS [12].

It is recommended that the general population should regularly participate in resistance training to increase MM and strength [13, 14]. However, currently, there are no specific guidelines for physical activity or exercise for post-BS patients, and therefore, the existing training protocols vary widely in type, intensity, duration, and frequency [15]. Although exercise has shown promising results in restoring MS in patients post-BS, more thorough research is still required to determine how effective these initiatives are [16]. Resistance training, in particular, has received recognition as a beneficial adjunct therapy to support post-BS recovery and specifically improve MS [11, 16]. Several studies [11, 17–23] have assessed the effects of exercise interventions on MS post-BS, utilizing various assessment tools such as 1 repetition maximum (1RM), handgrip strength, and repetitions until volitional exhaustion. These studies showed improvements in MS [11, 17–20, 22, 23] and physical function [11, 17, 21, 22]. However, a more recent study [24] has demonstrated that an intervention involving both exercise and nutritional behavior did not effectively promote improvements in MS. Given these conflicting evidence, further research is necessary to ascertain the optimal design of training programs and their impact on both absolute and relative MS in this population. The present study aims to bridge this knowledge gap by investigating the effect of an 11-month multicomponent exercise training program (MEP) on MS post-BS.

## Materials and Methods

### Study Design, Patient Recruitment, and Randomization

This is a secondary analysis of the registered open-label, single-center randomized controlled trial (ClinicalTrials.gov/NCT02843048) [25]. Local Hospital Ethics Committee approved the protocol (CES 192-14). Recruitment was carried out between April 2016 and November 2017. Inclusion criteria: age 18–65, BMI >35 kg.m-2, referral for primary Roux-en-Y gastric bypass (RYGB) or sleeve gastrectomy. Exclusion criteria: health conditions precluding exercise, active metabolic bone disease, perimenopausal state, pregnancy, or nursing, and revisional BS. Individuals with endocrine-related obesity were excluded. After agreeing to participate in the study, patients were randomized into a control group (CG) or exercise training group (EG) by minimization according to the following covariates: sex, age, BMI, type 2 diabetes, menopause, thiazide diuretics use, and smoking. The final allocation was unbalanced 1:2, favoring the EG, as previously described [25, 26]. Written informed consent from the patients was obtained.

### Interventions

#### Control Versus Exercise Groups

After BS, CG received standard care [27], which includes multivitamins (e.g., Centrum®), protein supplements (e.g., Protifar®, Fantomalt®), and verbal advice to increase physical activity; however, no structured exercise prescriptions were given. The EG, alongside standard care, underwent a 3-session/week, 75-min/session MEP for 11-month post-BS, as previously published [25, 26]. Sessions included warm-up (5 min), ground impact exercises (20 min), balance training (10 min), resistance training (35 min), and cooldown (5 min). Ground impact exercises involved high-force activities like runs and jumps. Balance drills challenged static and dynamic postural control. Resistance training covered major body regions with 2 to 3/sets of 4 to 12/reps at ≈ 65% to 85% of 1RM, adjusting loads individually. Adherence was tracked through attendance records.

### Measurements and Outcomes

Measurements were conducted at the local Research Centre. Baseline (1 to 3 month before surgery) and post-surgery assessments (1, 6, and 12 months) included absolute and relative knee/trunk extension/flexion MS, and MS relative to BW, total, and regional (trunk and thigh) LM.

#### Body Composition and Anthropometry Assessments

**Fig. 1:**
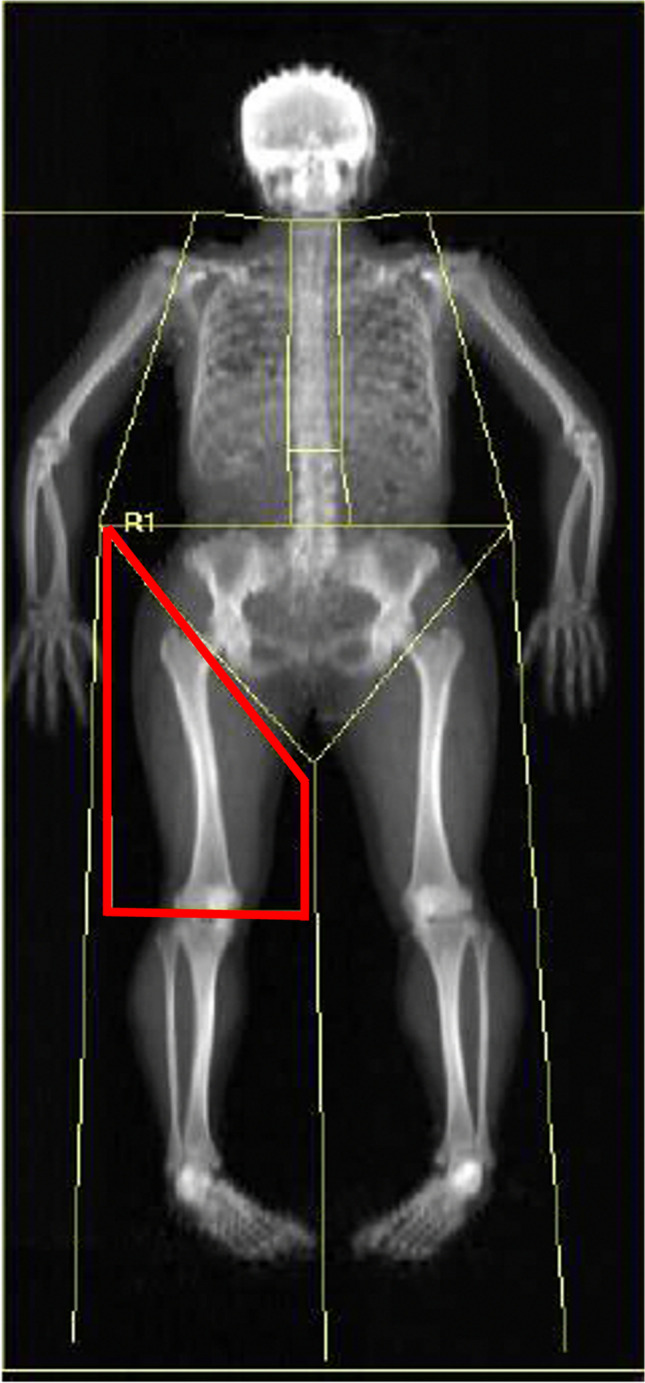
Thigh lean mass defined by DXA. Note: Region of interest created to analyze the lean mass of the thigh. This method facilitates the computation of lean mass contained within the designated area

DXA with the Hologic Explorer QDR measured total and regional LM (Hologic Inc, Bedford, MA, USA). Patients were measured in light clothes without metal jewelry or other metal items to ensure accuracy. Best procedures were followed for patient positioning in equipment [28]. LM was examined by whole-body scanning. In the whole-BC evaluation, the right upper limb was employed to bridge the gaps in the left upper limb due to the patient’s width [28]. Thigh LM was defined by a DXA subregion, as shown in Fig. 1. The total LM DXA coefficient of variation was 0.7%. All assessments were done by the same skilled specialist. A digital scale measured the patient’s BW (model 899, Seca, Hamburg, Germany).

#### Muscle Strength Assessment

Dominant lower-limb knee and trunk extension and flexion concentric MS were evaluated with an isokinetic dynamometer (Biodex System 4 Pro, Biodex Medical Systems, Shirley, NY, USA). The lower-limb MS test (quadriceps femoris/ hamstrings) was performed with the patient seated in the equipment chair, with the trunk and knees fixed by straps. The range of motion was 90° taking as reference the maximum knee extension (0°). The evaluation of the knee extension/flexion strength was performed continuously by concentric contractions (flexion/extension) at maximal intensity. Two angular velocities were used: (i) 60°/s and (ii) 180°/s. Each subject performed four and eight repetitions at the angular velocity 60°/s and 180°/s, respectively. The trunk MS test was conducted with the patient in a semi-sitting position (functional position) in a seat coupled to the dynamometer, with knees flexed 15° and the trunk and knees fixed by straps. The trunk range of motion was 70°, between 20° hyperextension and 50° flexion. Trunk extension/flexion strength evaluation was performed continuously by concentric contractions (flexion/extension) at maximal intensity. Two angular velocities were also employed for the trunk: (i) 60°/s and (ii) 120°/s. Four repetitions at the angular velocity of 60°/s and six repetitions at the angular velocity of 120°/s were performed. The windowing option was also applied to isolate and study specific portions of the movement, allowing for a more detailed analysis of strength, peak torque, power, or other relevant variables. This feature assisted in identifying any potential weaknesses, imbalances, or abnormalities during a particular phase of the movement.

### Adverse Events

Prior reports from this clinical trial [25, 26] have detailed data on urgent medical appointments for adverse events. The information was obtained from the patient’s national health system registry.

### Data Analysis

The primary outcome was the between-group difference in knee and trunk MS at 6- and 12-month post-BS expressed in absolute values (Nm). Primary intention-to-treat analysis was conducted by comparing outcomes between groups as randomized, using linear mixed models to examine the treatment effect. The treatment effect was defined as the estimated between-group differences at 6 months and at the end of the first year post-BS, accounting for any baseline differences. These models included group, time, and their interaction as fixed effects and the subjects as random effects. Baseline values of the dependent variable, BMI, and age were included as covariates, along with surgery type, menopause status, diabetes, and smoker status. Bonferroni correction was applied when necessary and the adjusted p-value was presented. A sub-analysis of the attendance rate in the training sessions (e.g., >50% vs CG) was also conducted. The treatment effect was the estimated between-group differences after 6-month and 1-year post-BS. Attendance rate, time, and their interaction were fixed effects, whereas individuals were random. The variables considered included the type of surgery, menopausal status, diabetes, and smoking status, as well as baseline BMI and age. A p-value corrected for Bonferroni adjustment was subsequently reported. Cohen’s *d* measured effect size: small = 0.2, medium = 0.5, big = 0.8. Statistical analysis were conducted with R (version 4.1.2, R Foundation for Statistical Computing, Vienna, Austria). The data are estimated marginal means (EMM) with confidence intervals (95%CI). Estimated mean difference (EMD) and 95% CI showed the exercise treatment effect. The significance level was set as = 0.05.

## Results

### Study Participants

**Fig. 2:**
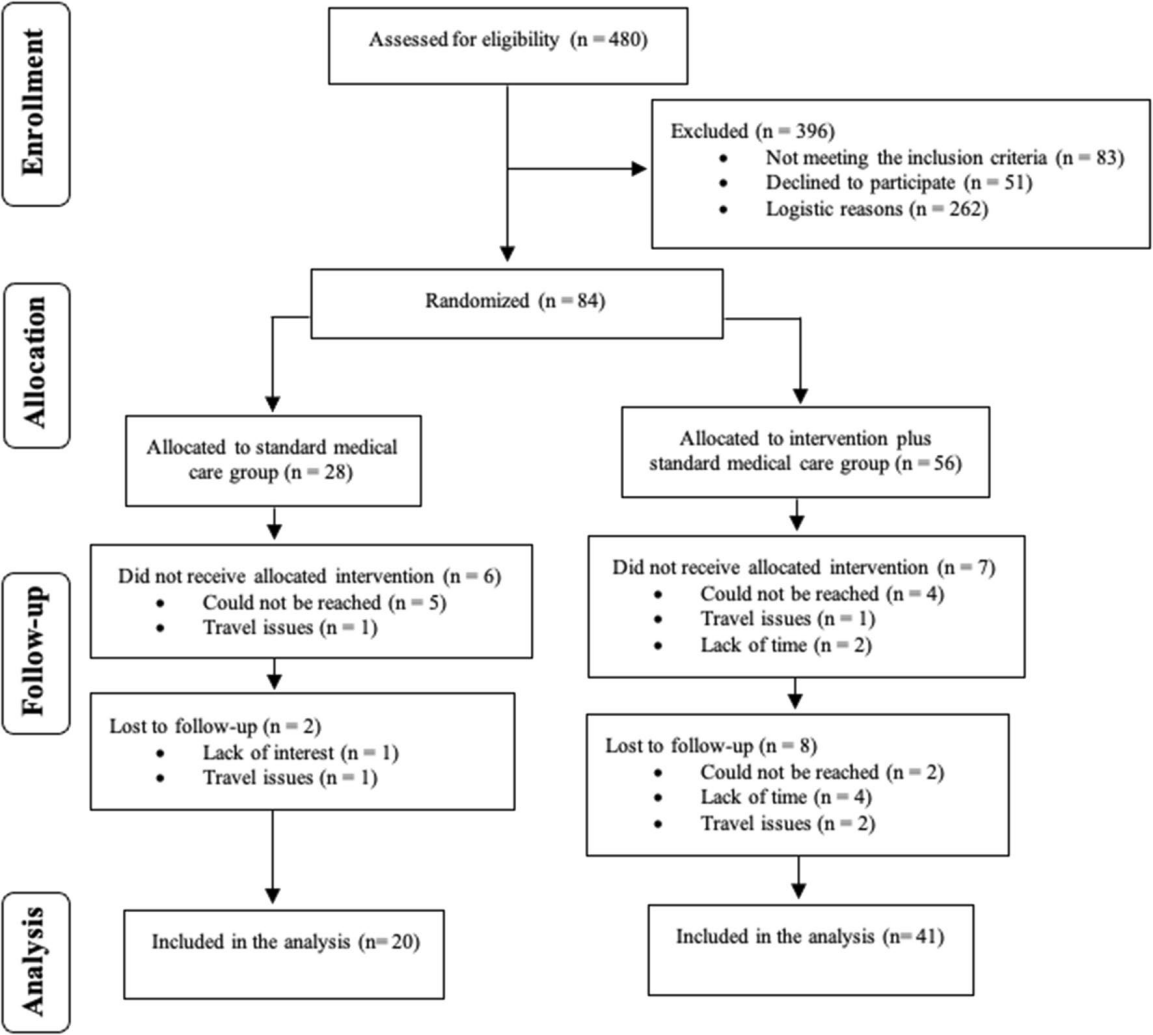
Flow of participant enrollment, allocation, follow-up, and analysis

Sixty-one of 84 participants completed assessments at 6- or 12-month post-BS (Fig. 2). Dropout rates were 29% for CG and 18% for EG. The final sample: 20 in CG, 41 in EG, mean age 43.2±10.1 years, BMI 44.1±4.6 kg∙m-2, 82% females, 24.6% with type 2 diabetes, 19.7% current smokers. EG attendance was 38%, with 25 participants with <50% attendance, Supplementary Table S1. Information was previously reported in studies [25, 26].

### Changes in Absolute Dominant Knee Extension and Flexion Muscle Strength After Bariatric Surgery

**Fig. 3:**
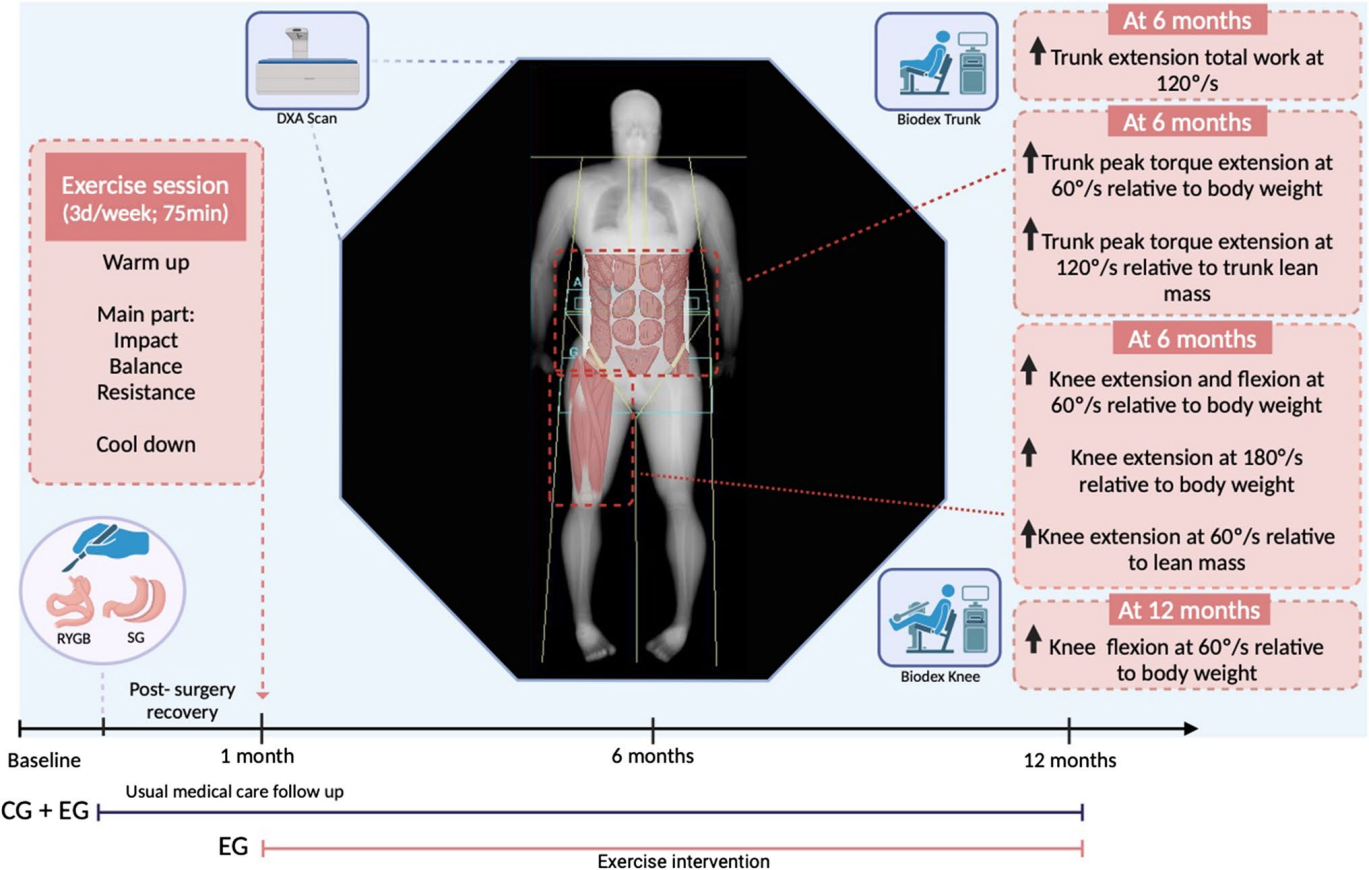
Chronic adaptations to 11-months of multicomponent exercise training after bariatric surgery. *Note*: Created with biorender.com (https://app.biorender.com/ (Accessed on 20 October 2023))

The MEP did not induce statistically significant effects on knee peak torque (PT-K) extension and flexion at 60°/s (*p* > 0.05) and PT-K extension and flexion at 180°/s (*p* > 0.05) at 6- or 12-month post-BS. Similarly, total work knee (TW-K) extension and flexion at 60° and 180° were not significantly improved by exercise at 6- or 12-month post-BS (*p* > 0.05). Time to peak torque knee (TPT-K) extension and flexion at 60°/s and 180°/s did not differ statistically between groups at 6- and 12-month post-BS (Table 1 and Fig. 3).

**Table 1:** Effects of a multicomponent exercise training on knee absolute muscle strength changes after bariatric surgery

Variable	Group	Pre-BS	1 month after BS	6 months after BS	12 months after BS	Treatment effect Baseline vs 6 months	Treatment effect baseline vs 12 months
Absolute knee muscle strength							
Knee PT extension 60°/s (Nm)	CG	154.4 (145; 163)	146 (138; 155)	138 (129; 147)	138 (128; 148)	2.9 (−6.6; 12.4), *p* = 0.553, *d* = −0.19	−2.5 (−13.4; 8.3), *p* = 0.646, *d =* 0.17
	EG	157 (150; 164)	138 (139; 154)	141 (134; 148)	135 (126; 143)		
Knee PT flexion 60°/s (Nm)	CG	91.2 (84.8; 97.7)	83.6 (77.2; 90.1)	82.2 (75.7; 88.6)	81.8 (74.7; 89.0)	2.7 (−4.2; 9.6), *p* = 0.449, *d =* −0.24	6.3 (−1.5; 0.1), *p* = 0.116, *d =* −0.57
	EG	90.0 (85.4; 94.7)	85.2 (80.4; 89.9)	84.8 (80.1; 89.6)	88.1 (83.0; 93.2)		
Knee PT extension 180°/s (Nm)	CG	117.3 (110.7; 124)	105.7 (99.1; 112)	98.7 (92.1; 105)	102.9 (95.6; 110)	2.6 (−9.6; 4.5), *p* = 0.480, *d =* −0.23	−3.6 (−11.6; 4.5), *p* = 0.386, *d =* 0.32
	EG	119.8 (114.5; 125)	106.6 (101.2; 112)	101.3 (95.9; 107)	99.3 (93.6; 105)		
Knee PT flexion 180°/s (Nm)	CG	65.1 (59.6; 70.6)	59.8 (54.3; 65.3)	58.5 (53.0; 64.0)	62.3 (56.1; 68.4)	2.5 (−3.6; 8.5), *p* = 0.428, *d =* −0.24	0.1 (−6.7; 7.0), *p* = 0.972, *d =* −0.012
	EG	65.9 (61.9; 69.9)	62.1 (58.0; 66.2)	60.9 (56.8; 65.0)	62.4 (58.0; 66.8)		
Knee extension total work 60°/s (J)	CG	154 (145; 163)	146 (138; 155)	138 (129; 147)	138 (128; 148)	2.9 (−6.6; 12.4), *p* = 0553, *d =* −0.19	−2.5 (−13.4; 8.3), *p* = 0.646, *d =* 0.17
	EG	157 (150; 164)	146 (139; 154)	141 (134; 148)	135 (128; 143)		
Knee flexion total work 60°/s (J)	CG	91.2 (84.8; 97.7)	83.6 (77.2; 90.1)	82.2 (75.7; 88.6)	81.8 (74.7; 89.0)	2.7 (−4.2; 9.6), *p* = 0.449, *d =* −0.24	6.3 (−1.5; 14.1), *p* = 0.116, *d =* −0.57
	EG	90.0 (85.4; 94.7)	85.2 (80.4; 89.9)	84.8 (80.1; 89.6)	88.1 (83.0; 93.2)		
Knee extension total work 180°/s (J)	CG	117.3 (110.7; 124)	105.7 (99.1; 112)	98.7 (92.1; 105)	102.9 (95.6; 110)	2.6 (−4.5; 9.6), *p* = 0.480, *d =* −0.40	−3.6 (−11.6; 4.5), *p* = 0.386, *d =* 0.12
	EG	119.8 (114.5; 125)	106.6 (101.2; 112)	101.3 (95.9; 107)	99.3 (93.6; 105)		
Knee flexion total work 180°/s (J)	CG	65.1 (59.6; 70.6)	59.8 (54.3; 65.3)	58.5 (53.0; 64.0)	62.3 (56.1; 68.4)	2.5 (−3.6; 8.5), *p* = 0.428, *d =* −0.24	0.1 (−6.8; 7.0), *p* = 0.972, *d =* −0.01
	EG	65.9 (61.9; 69.9)	62.1 (58.0; 66.2)	60.9 (56.8; 65.0)	62.4 (58.0; 66.8)		
Time to PT extension 60°/s (Mseg)	CG	540 (486; 594)	513 (459; 567)	512 (457; 566)	585 (523; 646)	45.2 (−17.3; 107.8), *p* = 0.158, *d =* −0.42	−36.4 (−107; 34.2), *p* = 0.315, *d =* 0.34
	EG	537 (499, 575)	540 (459; 567)	557 (518; 596)	548 (505; 591)		
Time to PT flexion 60°/s (Mseg)	CG	637 (578; 696)	522 (462; 581)	541 (481; 601)	502 (435; 569)	−43.5 (−111.5; 24.5), *p* = 0.211, *d =* 0.39	9.7 (−66.5; 86), *p* = 0.803, *d =* −0.09
	EG	596 (554; 638)	514 (472; 557)	497 (455; 540)	512 (465; 558)		
Time to PT extension 180°/s (Mseg)	CG	217 (198; 236)	234 (215; 253)	230 (211; 250)	229 (208; 250)	−12.2 (−33.9; 9.6), *p* = 0.274, *d =* 0.34	−5.6 (−30.0; 18.8), *p* = 0.651, *d =* 0.16
	EG	222 (209; 235)	207 (193; 221)	218 (205; 232)	223 (208; 238)		
Time to PT flexion 180°/s (Mseg)	CG	349 (275; 424)	376 (301; 450)	345 (269; 420)	336 (253; 420)	18.7 (66.6; 104.0), *p* = 0.668, *d =* −0.14	−21.8 (−116.9; 73.2), *p* = 0.653, *d =* 0.16
	EG	307 (254; 361)	336 (281; 390)	364 (309; 418)	315 (255; 374)		

Data are presented as estimated marginal mean (EMM) and 95% CI. Treatment effect was reported as estimated mean difference (EMD) and 95% CI. Statistical significance was considered when *p* < 0.05. Cohen’s *d =* (*d*)

*BS*, bariatric surgery; *CG*, control group; *EG*, exercise group, *PT*, peak torque

### Changes in Absolute Trunk Extension and Flexion Muscle Strength After Bariatric Surgery

The MEP has not induced statistically significant effects on absolute PT-T extension and flexion at 60°/s and 120°/s (*p* > 0.05) at 6- and 12-month post-BS. Similarly, TW-T extension and flexion at 60°/s, as well as flexion at 120°/s, were not improved by MEP participation (*p* > 0.05) at 6- and 12-month post-BS. While MEP showed a statistically significant effect on TW-T extension at 120°/s after 6 months (30.3 Nm; *p* = 0.024), this effect was not sustained at 12 months (*p* > 0.05). TPT-T extension and flexion at 60°/s and 120°/s also did not show improvement with MEP participation at 6- and 12-month post-BS (Table 2 and Fig. 3).

**Table 2:** Effects of a multicomponent exercise training on trunk absolute muscle strength changes after bariatric surgery

Variable	Group	Pre-BS	1 month after BS	6 months after BS	12 months after BS	Treatment effect Baseline vs 6 months	Treatment effect baseline vs 12 months
Absolute trunk muscle strength							
Trunk PT extension 60°/s (Nm)	CG	244 (221; 268)	216 (193; 240)	229 (204; 254)	264 (237; 292)	28.5(−10.1; 46.8); *p* = 0.206; *d =* −0.39	28.5 (−2.82; 59.9); *p* = 0.08; *d =* 0.61
	EG	239 (222; 255)	241 (193; 240)	247 (230; 265)	236 (217; 254)		
Trunk PT flexion 60°/s (Nm)	CG	99.8 (90.8; 108.8)	83.9 (74.9; 92.9)	74.7 (65.2; 84.1)	81.5 (71.2; 91.8)	−4.3 (−6.1; 14.7); *p* = 0.416; *d =* −0.28	0.75 (−10.7; 12.2), *p* = 0.898; *d =* −0.05
	EG	100.0 (93.0; 107.0)	80.4 (73.3; 87.6)	79.0 (72.0; 86.1)	82.3 (74.8; 89.7)		
Trunk PT extension 120°/s (Nm)	CG	163 (134; 192)	162 (133; 192)	149 (119; 180)	182 (149; 215)	−41.3 (7.0; 75.6); *p* = 0.184; *d =* −0.81	−3.4 (−34.0; 40.8); *p* = 0.859; *d =* −0.07
	EG	166 (145; 187)	180 (158; 202)	191 (168; 213)	186 (162; 209)		
Trunk PT flexion 120°/ (Nm)s	CG	67.7 (58.5, 76.9)	58.3 (48.7; 67.9)	49.3 (39.4; 59.2)	56.7 (46.1; 67.4)	6.6 (−3.8; 17.1); *p* = 0.217; *d =* −0.45	−1.8 (−9.6; 13.3); *p* = 0.752; *d =* −0.13
	EG	68.5 (61.3; 75.8)	55.2 (47.7; 62.7)	55.9 (48.4; 63.4)	58.6 (50.9; 66.3)		
Trunk extension total work 60°/s (J)	CG	190 (171; 209)	177 (158; 196)	179 (159; 200)	205 (183; 227)	9.7 (−13.4; 32.8); *p* = 0.410; *d =* −0.25	−16.3 (−41.9; 9.3); *p* = 0.213, *d =* 0.43
	EG	186 (172; 200)	191 (177, 105)	189 (175; 203)	189 (174; 204)		
Trunk flexion total work 60°/s (J)	CG	67.3 (60.9; 74.2)	58.7 (52.1, 65.4)	52.6 (45.6; 59.6)	56.9 (49.3; 64.4)	1.3 (−6.3; 8.9); *p* = 0.743; *d =* −0.11	2.6 (−5.8; 10.9); *p* = 0.547; *d =* −0.23
	EG	67.5 (62.4; 72.4)	55.1 (49.9; 60.3)	53.8 (48.7; 59.0)	59.4 (54.0; 64.9)		
Trunk extension total work 120°/s (J)	CG	109 (86.9; 131)	105 (82.2; 127)	102 (78.5; 125)	124 (98.6; 149)	30.3 (4.10; 56.4); ***p*** **= 0.024;** *d =* −0.80	8.9 (−19.72; 37.47); *p =* 0.544; *d =* −0.23
	EG	110 (93.3; 126)	124 (106.9; 140)	132 (115.2; 149)	133 (115.1; 150)		
Trunk flexion total work 120°/s (J)	CG	41.9 (35.5; 48.4)	33.3 (26.9; 39.8)	27.7 (20.9; 34.5)	32.2 (24.9; 39.5)	5.1 (−2.09; 12.26); *p =* 0.166; *d =* −0.48	3.8 (−4.08; 11.60); *p =* 0.348; *d =* −0.35
	EG	41.5 (36.6; 46.4)	31.8 (26.8; 36.8)	32.8 (27.8; 37.8)	36.0 (30.8; 41.2)		
Time to PT extension 60°/s (Mseg)	CG	456 (368; 543)	456 (369; 543)	385 (293; 477)	292 (191; 393)	−41.7 (147.5; 64.1); *p =* 0.441, *d =* 0.25	57.8 (−58.8; 174.4); *p =* 0.333; *d =* −0.34
	EG	445 (382; 507)	346 (282; 410)	343 (279; 407)	350 (281; 419)		
Time to PT extension 120°/s (Mseg)	CG	241 (216; 265)	239 (215; 264)	219 (194; 245)	227 (199; 255)	−20.6 (−50.4; 9.2); *p =* 0.177; *d =*	−21.9 (−54.5; 10.6); *p =* 0.188; *d =*
	EG	219 (201; 237)	208 (189; 226)	199 (180; 218)	205 (185; 225)		
Time to PT flexion 60°/s (Mseg)	CG	465 (324; 606)	626 (485; 767)	560 (412; 709)	598 (435; 761)	−9.5 (−178.3; 159.2); *p =* 0.912; *d =* 0.04	−51.2 (−237.0; 134.6); *p =* 0.590; *d =* 0.20
	EG	429 (327; 531)	543 (439; 647)	551 (447; 655)	547 (435; 659)		
Time to PT flexion 120°/s (Mseg)	CG	388 (341; 436)	434 (384; 484)	488 (437; 540)	437 (381; 493)	−10.7 (−68.3; 46.9); *p =* 0.717; *d =* 0.13	8.2 (−55.3; 71.7); *p =* 0.801; *d =* −0.10
	EG	378 (343; 413)	486 (450; 522)	478 (441; 514)	445 (407; 484)		

Data are presented as estimated marginal mean (EMM) and 95%CI. Treatment effect was reported as estimated mean difference (EMD) and 95%CI. Statistical significance was considered when *p* < 0.05, and Cohen’s *d =* (*d*)

*BS*, bariatric surgery; *CG*, control group; *EG*, exercise group, *PT*, peak torque

### Changes in Relative Dominant Knee Extension and Flexion Muscle Strength After Bariatric Surgery

Six months post-BS, exercise improved knee MS at PT-K extension at 60°/s relative to BW (0.14 Nm, *p* = 0.047), PT-K flexion at 60°/s relative to BW (0.07 Nm, *p* = 0.026), PT-K extension at 180°/s relative to BW (0.09 Nm, *p* = 0.009), PT-K extension at 60°/s relative to thigh LM (1.40 Nm, *p* = 0.040), and PT-K extension at 180°/s relative to BW (0.09 Nm, *p* = 0.009). Notably, only PT-K flexion at 60°/s relative to BW showed significant exercise-induced effects 12-month post-BS (0.08 Nm, *p* = 0.038) (Table 3 and Fig. 3).

**Table 3:** Effects of a multicomponent exercise training on relative knee muscle strength changes after bariatric surgery

Variable	Group	Pre-BS	1 month after BS	6 months after BS	12 months after BS	Treatment effect Baseline vs 6 months	Treatment effect baseline vs 12 months
Relative knee muscle strength							
Knee PT extension 60°/s Relative to BW (Nm∙kg ^– 1^)	CG	1.32 (1.23; 1.40)	1.34 (1.25; 1.42)	1.49 (1.40; 1.58)	1.67 (1.57; 1.77)	0.14 (0.05; 0.24); ***p =*** **0.047**; *d =* −0.95	0.048 (0.37; 0.59); *p =* 0400; *d =* −0.32
	EG	1.33 (1.27; 1.40)	1.37 (1.30; 1.44)	1.63 (1.57; 1.70)	1.72 (1.65; 1.79)		
Knee PT flexion 60°/s Relative to BW (Nm∙kg ^– 1^)	CG	0.69 (0.64; 0.75)	0.67 (0.66; 0.73)	0.81 (0.76; 0.87)	0.92 (0.85; 0.98)	0.07 (0.01; 0.14); ***p =*** **0.026**; *d =* −0.71	0.08 (0.01; 0.14); ***p =*** **0.038**; *d =* −0.75
	EG	0.70 (0.66; 0.74)	0.72 (0.67; 0.77)	0.89 (0.84; 0.93)	0.99 (0.95; 1.04)		
Knee PT extension 60°/s Relative to LM (Nm∙kg ^– 1^)	CG	2.73 (2.59; 2.88)	2.80 (2.66; 2.94)	2.70 (2.56; 2.85)	2.79 (2.63; 2.95)	−0.13 (−0.02; 0.30); *p =* 0.092; *d =* −0.53	−0.09 (−0.27; 0.09); *p =* 0.334, *d =* 0.35
	EG	2.79 (2.69; 2.89)	2.87 (2.76; 2.97)	2.84 (2.74; 2.95)	2.70 (2.59; 2.82)		
Knee PT flexion 60°/s Relative to LM (Nm∙kg ^– 1^)	CG	1.40 (1.31; 1.50)	1.39 (1.30; 1.49)	1.47 (1.37; 1.57)	1.50 (1.39; 1.61)	0.03 (−0.08; 0.14); *p =* 0563; *d =* −0.18	0.04 (−0.08; 0.16); *p =* 0.533; *p =* −0.22
	EG	1.42 (1.35; 1.49)	1.46 (1.39; 1.53)	1.50 (1.43; 1.57)	1.54 (1.46; 1.62)		
Knee PT extension 60°/s Relative to thigh LM (Nm∙kg ^– 1^)	CG	21.7 (20.5; 22.9)	19.9 (18.7; 21.1)	18.1 (16.9; 19.2)	18.3 (17.0; 19.6)	1.40 (0.07; 2.74); ***p =*** **0.040;** *d =* −0.68	0.05 (−1.45; 1−56), *p =* 0.944; *d =* 0.03
	EG	22.4 (21.5; 23.2)	20.6 (19.8; 21.5)	19.5 (18.6; 20.4)	18.2 (17.3; 19.2)		
Knee PT flexion 60°/s Relative to thigh LM (Nm∙kg ^– 1^)	CG	11.3 (10.4; 12.1)	11.5 (10.6; 12.3)	12.8 (12.0; 13.7)	13.3 (12.3; 14.3)	0.41 (−0.55; 1.37), *p =* 0.406; *d =* −0.28	0.69 (−0.39; 1.77), *p =* 0.209, *d =* −0.47
	EG	11.4 (10.8; 12.1)	12.0 (11.3; 12.6)	13.2 (12.6; 13.9)	14.0 (13.3; 14.7)		
Knee PT extension 180°/s Relative to BW (Nm∙kg ^– 1^)	CG	0.88 (0.82; 0.94)	0.87 (0.81; 0.93)	0.98 (0.92; 1.04)	1.16 (1.09; 1.22)	0.09 (0.02; 0.15), ***p =*** **0.009;** *d =* −0.87	−0.01 (−0.09; 0.06), *p =* 0.716; *d =* 0.13
	EG	0.90 (0.86; 0.95)	0.90 (0.85; 0.94)	1.07 (1.02; 1.11)	1.15 (1.10; 1.19)		
Knee PT flexion 180°/s Relative to BW (Nm∙kg ^– 1^)	CG	0.50 (0.45; 0.55)	0.50 (0.45; 0.54)	0.61 (0.56; 0.65)	0.69 (0.64; 0.75)	0.03 (−0.02; 0.08), *p =* 0.281; *d =* −0.34	0.02 (−0.04; 0.08), *p =* 0.531; *d =* −0.23
	EG	0.51 (0.47; 0.55)	0.51 (0.47; 0.54)	0.64 (0.60; 0.67)	0.71 (0.67; 0.75)		
Knee PT extension 180°/s Relative to LM (Nm∙kg ^– 1^)	CG	1.84 (1.74; 1.93)	1.82 (1.73; 1.92)	1.79 (1.70; 1.89)	1.89 (1.78; 2.00)	0.07 (−0.04; 0.17), *p =* 0.229; *d =* −0.39	−0.06 (−0.18; 0.06), *p =* 0.327; *d =* 0.36
	EG	1.88 (1.81; 1.95)	1.87 (1.80; 1.94)	1.86 (1.79; 1.93)	1.83 (1.75; 1.93)		
Knee PT flexion 180°/s Relative to LM (Nm∙kg ^– 1^)	CG	1.02 (0.95; 1.10)	1.02 (0.95; 1.10)	1.07 (1.00; 1.15)	1.13 (1.04; 1.22)	0.02 (−0.07; 0.11), *p =* 0.633; *d =* −0.15	−0.01 (−0.11; 0.08), *p =* 0.780; *d =* 0.10
	EG	1.05 (0.99; 1.10)	1.05 (0.99; 1.11)	1.09 (1.04; 1.15)	1.12 (1.05; 1.18)		
Knee PT extension 180°/s Relative to thigh LM (Nm∙kg ^– 1^)	CG	14.8 (13.9; 15.7)	15.0 (14.1; 15.9)	15.7 (14.8; 16.6)	16.9 (15.9; 18.0)	0.62 (−0.40; 1.65), *p =* 0.236; *d =* −0.40	−0.38 (−1.53; 0.77), *p =* 0.521; *d =* 0.25
	EG	15.2 (14.5; 15.9)	15.2 (14.5; 15.9)	16.3 (15.7; 17.0)	16.6 (15.8; 17.3)		
Knee PT flexion 180°/s Relative to thigh LM (Nm∙kg ^– 1^)	CG	8.20 (7.46; 8.94)	8.48 (7.75; 9.22)	9.39 (8.66; 10.13)	10.17 (9.36; 10.98)	0.23 (−0.58; 1.04) , *p =* 0.577; *d =* −0.19	−0.04 (−0.94; 0.87), *p =* 0.937; *d =* 0.03
	EG	8.43 (7.88; 8.97)	8.57 (8.02; 9.12)	9.62 (9.08; 10.17)	10.13 (9.54; 10.72)		

Data are presented as estimated marginal mean (EMM) and 95%CI. Treatment effect was reported as estimated mean difference (EMD) and 95%CI. Statistical significance was considered when *p* < 0.05, and Cohen’s *d =* (*d*)

*BS*, bariatric surgery; *CG*, control group; *EG*, exercise group, *BW*, body weight; *LM*, lean mass, *PT*, peak torque

### Changes in Relative Trunk Extension and Flexion Muscle Strength After Bariatric Surgery

Exercise improved PT-T extension at 60°/s relative to body weight (0.3 Nm, *p* = 0.05) and PT-T extension at 120°/s relative to trunk lean mass (1.71 Nm, *p* = 0.020) 6 months after bariatric surgery (BS). Notably, the CG showed a notably better response in PT-T extension at 60°/s relative to total lean mass (−0.7 Nm, *p* = 0.033) and PT-T extension at 60°/s relative to trunk LM (−1.6 Nm, *p* = 0.024) at 12-month post-BS. No significant differences were observed between groups for other assessed relative MS variables (Table 4 and Fig. 3).

**Table 4:** Effects of a multicomponent exercise training on trunk relative muscle strength changes after bariatric surgery

Variable	Group	Pre-BS	1 month after BS	6 months after BS	12 months after BS	Treatment effect Baseline vs 6 months	Treatment effect baseline vs 12 months
Relative trunk muscle strength							
Trunk PT extension 60°/s Relative to BW (Nm∙kg ^– 1^)	CG	2.2 (1.9; 2.4)	2.1 (1.8; 2.4)	2.7 (2.5; 3.0)	3.6 (3.2; 3.9)	0.3 (0.01; 0.64); *p =* **0.05**; *d =* −0.64	−0.3 (−0.68; 0.04); *p =* 0.082; *d =* 0.63
	EG	2.2 (2.0; 2.4)	2.4 (2.2; 2.6)	3.1 (2.9; 3.3)	3.2 (3.0; 3.4)		
Trunk PT extension 120° Relative to BW (Nm∙kg ^– 1^)	CG	0.61 (0.51; 0.71)	0.59 (0.48; 0.69)	0.60 (0.48; 0.71)	0.78 (0.66; 0.91)	0.09 (−0.04; 0.21); *p =* 0.168; *d =* −0.52	0.01(−013; 0.14); *p =* 0.939; *d =* 0.03
	EG	0.62 (0.54; 0.70)	0.54 (0.45; 0.62)	0.68 (0.60; 0.77)	0.78 (0.69; 0.87)		
Trunk PT flexion 60°/s Relative to BW (Nm∙kg ^– 1^)	CG	0.88 (0.79; 0.98)	0.82 (0.73; 0.92)	0.90 (0.80; 1.00)	1.06 (0.95; 1.17)	0.06 (−0.05; 0.17); *p =* 0.289; *d =* −0.37	0.05 (−0.07; 0.18); *p =* 0.398; *d =* −0.33
	EG	0.88 (0.81; 0.96)	0.80 (0.72; 0.87)	0.97 (0.89; 1.04)	1.11 (1.03; 1.19)		
Trunk PT flexion 120° Relative to BW (Nm∙kg ^– 1^)	CG	0.61 (0.51; 0.71)	0.59 (0.48; 0.69)	0.60 (0.48; 0.71)	0.78 (0.66; 0.91)	0.09 (−0.04; 0.21); *p =* 0.168; *d =* −0.52	0.01 (−0.13; 0.14); *d =* 0.939; *d =* 0.03
	EG	0.62 (0.54; 0.70)	0.54 (0.45; 0.62)	0.68 (0.60; 0.77)	0.78 (0.69; 0.87)		
Trunk PT extension 60°/s Relative to total LM (Nm∙kg ^– 1^)	CG	4.4 (3.9; 4.9)	4.3 (3.8; 4.7)	5.0 (4.4; 5.5)	5.9 (5.3; 6.5)	0.31 (−0.29; 0.91); *p =* 0.315; *d =* −0.31	−0.7 (−1.42;−0.06); *p =* **0.033**; *d =* 0.75
	EG	4.4 (4.0; 4.7)	4.8 (4.6; 5.3)	5.3 (4.9; 5.6)	5.2 (4.8; 5.5)		
Trunk PT extension 120° Relative to total LM (Nm∙kg ^– 1^)	CG	2.4 (2.0; 2.7)	2.3 (1.9; 2.7)	2.1 (1.7; 2.5)	2.4 (2.0; 2.8)	0.21 (−0.21; 0.64); *p =* 0.332; *d =* −0.36	0.01 (−0.46; 0.49); *p =* 0.956; *d =* 0.02
	EG	2.4 (2.2; 2.7)	2.2 (1.9; 2.5)	2.3 (2.0; 2.6)	2.4 (2.1; 2.7)		
Trunk PT flexion 60°/s Relative to total LM (Nm∙kg ^– 1^)	CG	1.8 (1.6, 2.0)	1.7 (1.5; 1.8)	1.6 (1.4; 1.8)	1.7 (1.5; 1.9)	0.05 (−0.15; 0.26); *p =* 0.627; *d =* −0.16	0.01 (−0.22; 0.24); *p =* 0.940; *d =* −0.03
	EG	1.8 (1.7; 2.0)	1.6 (1.5; 1.7)	1.7 (1.5, 1.8)	1.8 (1.6; 1.9)		
Trunk PT flexion 120° Relative to total LM (Nm∙kg ^– 1^)	CG	1.2 (1.0; 1.4)	1.1 (0.9; 1.3)	1.0 (0.8; 1.2)	1.2 (1.0; 1.4)	0.10 (−0.11; 0.32); *p =* 0.355; *d =* −0.35	0.01 (−0.23; 0.25); *p =* 0.961; *d =* −0.01
	EG	1.2 (0.9; 1.3)	1.1 (0.9; 1.2)	1.1 (1.0; 1.3)	1.2 (1.1; 1.4)		
Trunk PT extension 60°/s Relative to trunk LM (Nm∙kg ^– 1^)	CG	8.7 (7.8; 9.7)	8.6 (7.6; 9.6)	9.9 (8.8; 10.9)	11.7 (10.5; 12.9)	0.51 (−0.69; 1.72); *p =* 0.407; *d =* −0.26	−1.6 (−2.95; −0.22); *p =* **0.024**; *d =* 0.80
	EG	8.7 (8.0; 9.4)	9.8 (9.1; 10.5)	10.4 (9.7; 11.1)	10.1 (9.3; 10.9)		
Trunk PT extension 120° Relative to trunk LM (Nm∙kg ^– 1^)	CG	5.5 (4.3; 6.7)	6.1 (4.9; 7.4)	6.2 (4.9; 7.5)	7.8 (6.4; 9.3)	1.71 (0.28; 3.13); *p =* **0.020**; *d =* −0.84	0.13 (−1.73; 1.46); p 0.869; *d =* 0.07
	EG	5.8 (4.9; 6.7)	7.2 (6.2; 7.5)	7.9 (7.0; 8.8)	7.7 (6.7; 8.7)		
Trunk PT flexion 60°/s Relative to trunk LM (Nm∙kg ^– 1^)	CG	3.5 (3.2; 3.9)	3.3 (3.0; 3.7)	3.2 (2.8; 3.5)	3.4 (3.0; 3.8)	0.11 (−0.29; 0.50); *p =* 0.601; *d =* −0.18	0.02 (−0.43; 0.47); *p =* 0.923; *d =* −0.04
	EG	3.6 (3.4; 3.8)	3.2 (2.9; 3.5)	3.3 (3.0; 3.5)	3.4 (3.2; 3.7)		
Trunk PT flexion 120° Relative to trunk LM (Nm∙kg ^– 1^)	CG	2.4 (2.0; 2.7)	2.3 (1.9; 2.7)	2.1 (1.7; 2.5)	2.4 (2.0; 2.9)	0.21 (−0.21; 0.65); *p =* 0.323; *d =* −0.37	0.01 (−0.48; 0.49); *p =* 0.983; *d =* 0.01
	EG	2.4 (2.2; 2.7)	2.1 (1.9; 2.4)	2.3 (2.0; 2.6)	2.4 (2.1; 2.7)		

Data are presented as estimated marginal mean (EMM) and 95%CI. Treatment effect was reported as estimated mean difference (EMD) and 95%CI. Statistical significance was considered when *p* < 0.05, and Cohen’s *d =* (*d*)

*BS*, bariatric surgery; *CG*, control group; *EG*, exercise group, *BW*, body weight; *LM*, lean mass, *PT*, peak torque

### Sub-analysis of Multicomponent Exercise Training Attendance Effects in Absolute and Relative Knee and Trunk Muscle Strength Changes Post-BS

Participation in multicomponent exercise training with an attendance rate >50% demonstrated notable improvements in absolute and relative MS post-BS (Supplementary Tables S2 to S5). At 6-month post-BS, participants with an attendance rate >50% showed significant improvements in PT-T extension at 60°/s (36.2 Nm, *p* = 0.036) and 120°/s (42.7 Nm, *p* = 0.044), TPT-K extension at 60°/s (99.4 Mseg, *p* = 0.017), PT-K extension at 60°/s relative to BW (0.2 Nm, *p* = 0.001), PT-K flexion at 60°/s relative to BW (0.1Nm, *p* = 0.012), PT-K extension at 60°/s relative to LM (0.2 Nm, *p* = 0.017), PT-K extension at 60°/s relative to thigh LM (2.4 Nm, *p* = 0.004), and PT-K extension at 180°/s relative to BW (0.1 Nm, *p* = 0.006). At 12-month post-BS, participants with an attendance rate >50% also exhibited significant enhancement in PT-K flexion at 60°/s relative to BW (0.1 Nm, *p* = 0.009). Moreover, at 6-month post-BS, significant improvements were observed in PT-T extension at 60°/s relative to BW (0.50 Nm, *p* = 0.010), PT-T flexion at 60°/s relative to BW (0.14 Nm, *p* = 0.047), and PT-T extension at 120° relative to trunk LM (1.93 Nm, *p* = 0.029).

## Discussion

This study investigated the impact of 11 months of MEP on MS post-BS. Our study found, in an intention to treat analysis, no significant effects of MEP on knee and trunk MS in extension and flexion at 6- and 12-month post-BS (60°/s and 180°/s, respectively). No statistically significant effects of exercise on TW-K and TPT-K extension and flexion at 60°/s and 180°/s were also detected. Six months post-BS, exercise significantly improved knee extension and flexion relative to BW and LM. At 6-month post-BS, MEP improved TW-T and PT-T extensions relative to BW. Our findings post-BS revealed MS significantly decreased, with knee extension at 60°/s reducing by 17.9%, flexion at 60°/s by 10%, and extension at 180°/s by 17.3%. Given the extent of these changes, exercise cannot prevent absolute MS decline. Hue and colleagues [29] found that 1-year post-BS, maximum MS decreased, notably in antigravitational muscles like the knee extensors (33.5% decrease). Lower-limb and upper-limb declines occurred. Given its importance as a marker of functional ability, cardiovascular health, and mortality risk, this absolute MS decline is concerning [30]. Patients post-BS face not only a decrease in absolute MS [31, 32] but higher fall risk, decreased physical function, and difficulty with daily tasks [20, 29].

Most research shows that supervised resistance training improves MS and physical function, boosting functional capacity and daily living activities post-BS [11]. Thus, post-BS patients should include resistance exercises in their training [33]. Studies [18, 34, 35] have shown that resistance exercise effectively improves absolute MS post-BS. Daniels and colleagues [18] observed that resistance training enhanced absolute MS and muscle quality in women without changing fat-free mass or muscle cross-sectional area after Roux-en-Y gastric bypass. Mundbjerg et al. [34] found that 26 weeks of concurrent supervised exercise increased hip abduction in absolute MS. Other studies [35, 36] have shown that the combination of resistance exercise with protein intake supplementation [36] can further mitigate MS loss post-BS [35] by favoring exercise-induced increases in MM [36]. In opposition, our results showed that the MEP protocol implemented in this study did not induce significant improvements in absolute MS at either the lower limbs or trunk.

MS can be considered in both absolute and relative terms [32]. In this regard, relative MS, which is expressed relative to BW or LM, better reflects functional capacity, physical performance, and muscle functional quality [32]. Although our results showed that participation in a MEP did not lead to improvements in absolute MS, significant benefits on both trunk and lower-limb relative MS were identified. Regarding MS relative to BW at 6-month post-BS, improvements were observed for knee extension and flexion at both lower (60^o^/s) and higher speeds (180^o^/s) of testing and only at higher speeds for knee extension. Importantly, improvements in knee flexion at 60°/s relative to BW were maintained at 12-month post-BS.

In line with our findings, a study [17] suggested that 6 months of exercise training in post-BS patients was also able to improve MS relative to BW compared to a CG (2.4kg/BW vs. 1.4kg/BW). Furthermore, improvements in both knee extension and flexion strength after exercise training appear to significantly contribute to static and dynamic balance improvements [37]. Data from older adults have also shown that relative MS, especially in the lower extremities, is an important predictor of falls and injury in this population [38]. MS is a crucial determinant of overall health, well-being, mobility, and injury prevention [30, 39–42]. A higher relative MS also contributes to better cardiovascular health and lower mortality risk [30, 42].

Specific MS is expressed relative to MM and reflects the muscle contractile efficiency [43, 44]. In addition to improvements in MS relative to BW, our results show that exercise also effectively improved specific strength of knee extension at 60°/s as well as trunk extension at 120°/s at 6-month post-BS. These improvements were, nonetheless, not maintained at 12-month post-BS. Several studies have investigated the effects of exercise post-BS [11, 17–23, 35, 45, 46]. Of these, two studies [18, 35] showed improvements in lower-limb-specific MS compared with pre-surgery, ranging from +12% [35] to +36% [18]. Stegen et al. [20] showed that an exercise program with 12 weeks duration including both strength and endurance, starting 1-month post-BS, increased quadriceps-specific strength by 72% and hamstrings-specific strength by 27%. These findings agree with our results despite the magnitude of the specific muscle increments in our sample was lower.

The disparities between our findings and those of prior studies may be attributed to methodological distinctions, including sample and intervention characteristics. For instance, in our study, only women were included and the time of intervention onset post-bariatric surgery differed from previous studies [47] as well as intervention duration [48], exercise training protocol [48], and associated therapeutical interventions such as dietary supplementation with protein [26, 49]. In addition, the majority of the studies cited employed various tools to assess MS, including 1 repetition maximum (1RM) [11, 17–20], 10RM [23], handgrip [21, 35, 45, 46], and repetitions until exhaustion [22] which hinders a direct comparison of the results between ours and other studies.

This study contains limitations. Most important was the low EG attendance. As evidenced by our findings, the CG demonstrated notably better responses in MS compared to the EG, particularly in PT-T extension at 60°/s relative to total lean mass and trunk lean mass. Attendance rates varied over different intervals: from 1 month to 6 months post-BS, it was 50.5%; from 1 month to 12 months, it was 38%; and from 6 months to 12 months, it decreased to 27% However, longer-term interventions have showed high variability and low attendance rates [50]. Another limitation was the lack of nutritional control which could have biased the MM and strength findings. Moreover, the lack of a power analysis for the current sample size concerning the reported muscle strength outcomes is acknowledged as an additional limitation. Nevertheless, this was a secondary analysis of the data and the use of post-hoc power analysis is highly controversial. The study’s strengths include employing DXA to assess BC for thigh LM and an isokinetic dynamometer to evaluate MS. Both methods are gold standards for BC and MS evaluation. To the authors’ knowledge, this is the first research to compare knee flexion/extension to thigh LM. The MEP plan included cardiorespiratory and strength training, and both groups received intensive intervention and follow-up. Further research is necessary to identify the most effective exercise program for enhancing MS following BS, including interventions aimed at promoting exercise adherence and exploring the impact of different exercise intensities and frequencies.

## Conclusions

The findings of this study suggest that a multicomponent exercise training program may not be sufficient to induce significant improvements in absolute MS for the lower limb and trunk post-BS. However, the exercise program may be effective in improving several relative MS parameters, especially in the lower-limb region, at least in the medium term after surgery. The lack of significant effects on absolute MS may be related to the reduced MM and strength typically observed in patients with obesity post-BS and to the difficulty in promoting MM gains in the context of extreme calorie restriction as is the case post-BS.

### Supplementary Information

Supplementary file 1 (DOCX 15 kb)

Supplementary file 2 (DOCX 25 kb)

Supplementary file 3 (DOCX 23 kb)

Supplementary file 4 (DOCX 23 kb)

Supplementary file 5 (DOCX 23 kb)

## Data Availability

Participant data are available upon reasonable request from the corresponding author. Requests should be reasonable and accompanied by research proposals that have received appropriate ethical approval. Data will be made available in an anonymized format in compliance with applicable privacy and data protection laws.

## References

[1] Blüher M (2019). Obesity: global epidemiology and pathogenesis. Nat Rev Endocrinol.

[2] Puzziferri N (2014). Long-term follow-up after bariatric surgery: a systematic review. JAMA.

[3] Mulla CM, Middelbeek RJW, Patti ME (2018). Mechanisms of weight loss and improved metabolism following bariatric surgery. Ann N Y Acad Sci.

[4] Maïmoun L (2019). Acute and longer-term body composition changes after bariatric surgery. Surg Obes Relat Dis.

[5] King WC (2018). Comparison of the performance of common measures of weight regain after bariatric surgery for association with clinical outcomes. Jama.

[6] Switzer NJ (2016). Revisional bariatric surgery. Surg Clin North Am.

[7] Dulloo AG (2021). Physiology of weight regain: lessons from the classic Minnesota Starvation Experiment on human body composition regulation. Obes Rev.

[8] Volaklis KA, Halle M, Meisinger C (2015). Muscular strength as a strong predictor of mortality: a narrative review. Eur J Intern Med.

[9] Morgan PT, Smeuninx B, Breen L. Exploring the impact of obesity on skeletal muscle function in older age. Front Nutr. 2020;7:569904. 10.3389/fnut.2020.569904. 10.3389/fnut.2020.569904 PMC7736105 33335909

[10] Donini LM (2020). Critical appraisal of definitions and diagnostic criteria for sarcopenic obesity based on a systematic review. Clin Nutr.

[11] Huck CJ (2015). Effects of supervised resistance training on fitness and functional strength in patients succeeding bariatric surgery. J Strength Cond Res.

[12] Reinmann A, Gafner SC, Hilfiker R, et al. Bariatric surgery: consequences on functional capacities in patients with obesity. Front Endocrinol (Lausanne). 2021;12:646283. 10.3389/fendo.2021.646283. 10.3389/fendo.2021.646283 PMC8049139 33868175

[13] Hansen D (2020). Towards optimized care after bariatric surgery by physical activity and exercise intervention: a review. Obes Surg.

[14] Hughes DC, Ellefsen S, Baar K. Adaptations to endurance and strength training. Cold Spring Harb Perspect Med. 2018;8(6):a029769. 10.1101/cshperspect.a029769. 10.1101/cshperspect.a029769 PMC5983157 28490537

[15] Tabesh MR (2019). Nutrition, physical activity, and prescription of supplements in pre- and post-bariatric surgery patients: a practical guideline. Obes Surg.

[16] Vieira FT (2022). Effect of physical exercise on muscle strength in adults following bariatric surgery: a systematic review and meta-analysis of different muscle strength assessment tests. PLoS One.

[17] Gil S (2021). A randomized clinical trial on the effects of exercise on muscle remodelling following bariatric surgery. J Cachexia Sarcopenia Muscle.

[18] Daniels P (2017). Effect of a randomised 12-week resistance training programme on muscular strength, cross-sectional area and muscle quality in women having undergone Roux-en-Y gastric bypass. J Sports Sci.

[19] Hassannejad A (2017). The effect of aerobic or aerobic-strength exercise on body composition and functional capacity in patients with BMI ≥35 after bariatric surgery: a randomized control trial. Obes Surg.

[20] Stegen S (2011). Physical fitness in morbidly obese patients: effect of gastric bypass surgery and exercise training. Obes Surg.

[21] Herring LY, Stevinson C, Carter P, et al. The effects of supervised exercise training 12–24 months after bariatric surgery on physical function and body composition: a randomised controlled trial. Int J Obes (Lond). 2017;41(6):909–16. 10.1038/ijo.2017.60. 10.1038/ijo.2017.60 28262676

[22] Coleman KJ (2017). Understanding the capacity for exercise in post-bariatric patients. Obes Surg.

[23] Campanha-Versiani L (2017). The effect of a muscle weight-bearing and aerobic exercise program on the body composition, muscular strength, biochemical markers, and bone mass of obese patients who have undergone gastric bypass surgery. Obes Surg.

[24] Jassil FC (2023). Impact of nutritional-behavioral and supervised exercise intervention following bariatric surgery: the BARI-LIFESTYLE randomized controlled trial. Obesity.

[25] Diniz-Sousa F (2021). The effect of an exercise intervention program on bone health after bariatric surgery: a randomized controlled trial. J Bone Miner Res.

[26] Boppre G (2023). Effects of a supervised exercise training on body composition after bariatric surgery: a randomized controlled trial. Obesity.

[27] Mechanick JI (2013). Clinical practice guidelines for the perioperative nutritional, metabolic, and nonsurgical support of the bariatric surgery patient--2013 update: cosponsored by American Association of Clinical Endocrinologists, the Obesity Society, and American Society for Metabolic & Bariatric Surgery. Endocr Pract.

[28] Bazzocchi A (2016). DXA: Technical aspects and application. Eur J Radiol.

[29] Hue O (2008). Muscle force and force control after weight loss in obese and morbidly obese men. Obes Surg.

[30] Artero EG (2012). Effects of muscular strength on cardiovascular risk factors and prognosis. J Cardiopulm Rehabil Prev.

[31] Handrigan G (2010). Weight loss and muscular strength affect static balance control. Int J Obes.

[32] Alba DL (2019). Changes in lean mass, absolute and relative muscle strength, and physical performance after gastric bypass surgery. J Clin Endocrinol Metab.

[33] van Baak MA (2021). Effect of different types of regular exercise on physical fitness in adults with overweight or obesity: systematic review and meta-analyses. Obes Rev.

[34] Mundbjerg LH, Stolberg CR, Bladbjerg EM, et al. Effects of 6 months supervised physical training on muscle strength and aerobic capacity in patients undergoing Roux-en-Y gastric bypass surgery: a randomized controlled trial. Clin Obes. 2018;8(4):227–35. 10.1111/cob.12256. 10.1111/cob.12256 29896844

[35] Oppert JM (2018). Resistance training and protein supplementation increase strength after bariatric surgery: a randomized controlled trial. Obesity (Silver Spring).

[36] Lamarca F (2021). Effects of resistance training with or without protein supplementation on body composition and resting energy expenditure in patients 2-7 years postRoux-en-Y gastric bypass: a controlled clinical trial. Obes Surg.

[37] Marques EA (2017). Are resistance and aerobic exercise training equally effective at improving knee muscle strength and balance in older women?. Arch Gerontol Geriatr.

[38] Sadaqa M (2023). Effectiveness of exercise interventions on fall prevention in ambulatory community-dwelling older adults: a systematic review with narrative synthesis. Front Public Health.

[39] Sano Y (2018). An easy and safe training method for trunk function improves mobility in total knee arthroplasty patients: a quasi-randomized controlled trial. PLoS One.

[40] Granacher U (2013). The importance of trunk muscle strength for balance, functional performance, and fall prevention in seniors: a systematic review. Sports Med.

[41] Adams M (2023). Effects of physical activity interventions on strength, balance and falls in middle-aged adults: a systematic review and meta-analysis. Sports Med Open.

[42] Ruiz JR (2008). Association between muscular strength and mortality in men: prospective cohort study. Bmj.

[43] Barbat-Artigas S (2012). How to assess functional status: a new muscle quality index. J Nutr Health Aging.

[44] Valenzuela PL (2020). Obesity-associated poor muscle quality: prevalence and association with age, sex, and body mass index. BMC Musculoskelet Disord.

[45] de Oliveira Júnior GN (2021). Home-based exercise training during COVID-19 pandemic in post-bariatric patients: a randomized controlled trial. Obes Surg.

[46] Gallé F (2020). An exercise-based educational and motivational intervention after surgery can improve behaviors, physical fitness and quality of life in bariatric patients. PLoS One.

[47] Boppre G, Diniz‐Sousa F, Veras L, et al. Can exercise promote additional benefits on body composition in patients with obesity after bariatric surgery? A systematic review and meta-analysis of randomized controlled trials. Obes Sci Pract. 2021;1–12. 10.1002/osp4.542. 10.1002/osp4.542 PMC8804945 35127127

[48] Boppre G, Diniz-Sousa F, Veras L, et al. Does exercise improve the cardiometabolic risk profile of patients with obesity after bariatric surgery? A systematic review and meta-analysis of randomized controlled trials. Obes Surg. 2022;32(6):2056–68. 10.1007/s11695-022-06023-x. 10.1007/s11695-022-06023-x 35332396

[49] Mechanick JI (2020). Clinical practice guidelines for the perioperative nutrition, metabolic, and nonsurgical support of patients undergoing bariatric procedures - 2019 update: cosponsored by American Association of Clinical Endocrinologists/American College of Endocrinology, The Obesity Society, American Society for Metabolic and Bariatric Surgery, Obesity Medicine Association, and American Society of Anesthesiologists. Obesity (Silver Spring).

[50] Coen PM (2015). Clinical trial demonstrates exercise following bariatric surgery improves insulin sensitivity. J Clin Invest.

