# Supplementary material for: Impact of a Multicomponent Exercise Training Program on Muscle Strength After Bariatric Surgery: A Randomized Controlled Trial
Source: Obes Surg. 2024 Mar 27;34(5):1704–16. doi: 10.1007/s11695-024-07173-w (PMC11031478; doi:10.1007/s11695-024-07173-w)
Supplement: Supplementary file 1 — (DOCX 15 kb) [file 11695_2024_7173_MOESM1_ESM.docx]

**Supplementary Table S1:** Demographic, anthropometric, and clinical characteristics of the patients randomized to the control and exercise groups before bariatric surgery

| Variables | CG (n = 20) | EG (n = 41) |
| --- | --- | --- |
| Age (years) | 46.5 ± 8.5 | 41.6 ± 10.0 |
| Sex (female, %) | 80.0% | 84.8% |
| Surgery (RYGB, %) | 80.0% | 65.2% |
| Height (m) | 1.58 ± 0.09 | 1.59 ± 0.10 |
| Weight (kg) | 114.8 ± 15.4 | 110.1 ± 17.2 |
| Body mass index (kg⋅m^-2^) | 46.1 ± 4.2 | 43.0 ± 4.4 |
| Waist circumference (cm) | 126.5 ± 10.6 | 122.0 ± 13.3 |
| Hip circumference (cm) | 134.7 ± 9.0 | 130.7 ± 8.8 |
| Menopause (%) | 25.0% | 12.2% |
| Diabetes (%) | 20.0% | 26.8% |
| Current smoker (%) | 15% | 22% |

Note: Data are presented as Mean ± SD, and percentage (%)

Abbreviations: CG = control group; EG = exercise group; RYGB = Roux-en-Y gastric bypass.

The preceding information has already been reported in studies previously published [22, 23].
