# Supplementary material for: Impact of a Multicomponent Exercise Training Program on Muscle Strength After Bariatric Surgery: A Randomized Controlled Trial
Source: Obes Surg. 2024 Mar 27;34(5):1704–16. doi: 10.1007/s11695-024-07173-w (PMC11031478; doi:10.1007/s11695-024-07173-w)
Supplement: Supplementary file 2 — (DOCX 25 kb) [file 11695_2024_7173_MOESM2_ESM.docx]

Supplementary Table S2: Effects of a multicomponent exercise training attendance in absolute knee muscle strength changes post-BS

| Variable | Group | Pre-BS | 1-month post-BS | 6-months post-BS | 12-months post-BS | Treatment effect  Baseline vs 6-months | Treatment effect baseline vs 12-months |
| --- | --- | --- | --- | --- | --- | --- | --- |
| **Absolute muscle strenght** |  |  |  |  |  |  |  |
| Knee PT extension 60º/s (Nm∙kg ^– 1^) | CG | 154 (145; 163) | 146 (138; 155) | 138 (129; 147) | 138 (128; 148) | 7.1 (-4.6; 18.7), p = 0.237, d = -0.47 | -2.3 (-15.2; 10.6), p = 0.726, d = 0.15 |
|  | >50% | 157 (147; 167) | 147 (137; 157) | 145 (135; 155) | 136 (125; 146) |  |  |
| Knee PT flexion 60º/s (Nm∙kg ^– 1^) | CG | 91.3 (84.8; 97.8) | 83.7 (77.2; 90.2) | 82.3 (75.8; 88.8) | 81.9 (74.8; 89.1) | 5.6 (-2.9; 14.1), p = 0.193, d = -0.52 | 8.3 (-0.91; 17.5), p = 0.082, d = -0.76 |
|  | >50% | 88.7 (82.0; 95.5) | 82.1 (75.4; 88.9) | 87.9 (81.2; 94.6) | 90.2 (83.0; 97.3) |  |  |
| Knee PT extension 180º/s (Nm∙kg ^– 1^) | CG | 117.3 (110.7; 124) | 105.7 (99.0; 112) | 98.7 (92.1; 105) | 102.9 (95.6; 110) | 4.9 (-3.8; 13.6), p = 0.270, d = -0.44 | -3.5 (-13.8; 6.1), p = 0.479, d = 0.31 |
|  | >50% | 120.2 (112.8; 128) | 105.7 (98.4; 113) | 103.6 (96.2; 111) | 99.4 (91.7; 107) |  |  |
| Knee PT flexion 180º/s (Nm∙kg ^– 1^) | CG | 65.2 (59.6; 70.7) | 59.8 (54.3; 65.4) | 58.5 (53.0; 64.1) | 62.3 (56.3; 68.5) | 3.2 (-4.2; 10.6), p = 0.400, d = -0.31 | 0.1 (-8.3; 8.2), p = 0.990, d = 0.01 |
|  | >50% | 64.8 (58.9; 70.7) | 59.1 (53.2; 65.1) | 61.7 (55.8; 67.6) | 62.3 (56.0; 68.6) |  |  |
| Knee extension total work 60º/s (J) | CG | 154 (145; 163) | 146 (138; 155) | 138 (129; 146) | 138 (128; 148) | 7.1 (-4.6; 18.7), p = 0.237, d = -0.47 | -2.3 (-15.2; 10.6), p = 0.726, d = 0.15 |
|  | >50% | 157 (147; 167) | 147 (137; 157) | 145 (135; 155) | 136 (125; 146) |  |  |
| Knee flexion total work 60º/s (J) | CG | 91.3 (84.8; 97.8) | 83.7 (77.2; 90.2) | 82.3 (75.8; 88.8) | 81.9 (74.8; 89.1) | 5.6 (-2.9; 14.1), p = 0.193, d = -0.73 | 8.3 (-0.95; 17.6), p = 0.082, d = -0.76 |
|  | >50% | 88.7 (82.0; 95.5) | 82.1 (75.4; 88.9) | 87.9 (81.2; 94.6) | 90.2 (83.0; 97.3) |  |  |
| Knee extension total work 180º/s (J) | CG | 117.3 (110.7; 124) | 105.7 (99.0; 112) | 98.7 (92.1; 105) | 102.9 (95.6; 110) | 2.6 (-4.5; 9.6), p = 0.480, d = -0.44 | -3.6 (-11.6; 4.5), p = 0.386, d = 0.31 |
|  | >50% | 120.2 (112.8; 128) | 105.7 (98.4; 113) | 103.6 (96.2; 111) | 99.4 (91.7; 107) |  |  |
| Knee flexion total work 180º/s (J) | CG | 65.2 (59.6; 70.7) | 59.8 (54.3; 65.4) | 58.5 (53.0; 64.1) | 62.3 (56.2; 68.5) | 3.2 (-4.2; 10.6), p = 0.400, d = -0.37 | -0.1 (-8.3; 8.2), p = 0.990, d = 0.01 |
|  | >50% | 64.8 (58.9; 70.7) | 59.1 (53.2; 65.1) | 61.7 (55.8; 67.6) | 62.3 (56.0; 68.6) |  |  |
| Time to PT extension 60º/s (Mseg) | CG | 540 (487; 592) | 513 (460; 565) | 511 (457; 565) | 584 (524; 645) | 15.8 (-57.9; 89.5), p = 0.676, d = -0.14 | 99.4 (-180.4; -18,5), **p = 0.017**, d = 0.94 |
|  | >50% | 551 (495, 608) | 552 (496; 608) | 527 (471; 583) | 485 (425; 545) |  |  |
| Time to PT flexion 60º/s (Mseg) | CG | 637 (577; 697) | 522 (462; 581) | 541 (480; 601) | 502 (435; 570) | -36.1 (-118.2; 46.1), p = 0.390, d = 0.33 | 11.2 (-79.0; 101.3), p = 0.809, d = -0.10 |
|  | >50% | 586 (524; 649) | 534 (471; 597) | 505 (442; 567) | 513 (447; 580) |  |  |
| Time to PT extension 180º/s (Mseg) | CG | 217 (199; 236) | 234 (216; 253) | 230 (211; 249) | 229 (208; 250) | -15.4 (-41.3; 10.4), p = 0.552, d = 0.44 | -26.0 (-54.2; 2.2), p = 0.073, d = 0.74 |
|  | >50% | 229 (209; 249) | 207 (187; 227) | 215 (195; 235) | 203 (182; 224) |  |  |
| Time to PT flexion 180º/s (Mseg) | CG | 349 (274; 424) | 376 (301; 451) | 345 (269; 421) | 337 (252; 421) | 20.9 (-81.8; 123.6), p = 0.690, d = -0.14 | 17.3 (-116.9; 73.2), p = 0.653, d = 0.16 |
|  | >50% | 300 (221; 379) | 328 (249; 407) | 366 (287; 445) | 354 (270; 437) |  |  |
| Note: Data are presented as estimated marginal mean (EMM) and 95% CI. Treatment effect was reported as estimated mean difference (EMD) and 95% CI. Statistical significance was considered when p < 0.05. Cohen’s d = (d).  Abbreviations: BS= bariatric surgery; CG= control group; >50%= exercise group , PT= peak torque. | | | | | | | |
