# Supplementary material for: Impact of a Multicomponent Exercise Training Program on Muscle Strength After Bariatric Surgery: A Randomized Controlled Trial
Source: Obes Surg. 2024 Mar 27;34(5):1704–16. doi: 10.1007/s11695-024-07173-w (PMC11031478; doi:10.1007/s11695-024-07173-w)
Supplement: Supplementary file 3 — (DOCX 23 kb) [file 11695_2024_7173_MOESM3_ESM.docx]

| Variable | Group | Pre-BS | 1-month post-BS | 6-months post-BS | 12-months post-BS | Treatment effect  Baseline vs 6-months | Treatment effect baseline vs 12-months |
| --- | --- | --- | --- | --- | --- | --- | --- |
| **Absolute trunk muscle strenght** |  |  |  |  |  |  |  |
| Trunk PT extension 60º/s (Nm∙kg ^– 1^) | CG | 244 (221; 268) | 216 (193; 240) | 229 (205; 254) | 265 (238; 292) | 36.2 (2.5; 70.0); p = **0.036**; d = -0.78 | -21.0 (-56.5; 14.5); p = 0.249; d = 0.45 |
|  | > 50% | 234 (209; 258) | 239 (214; 263) | 265 (240; 291) | 244 (218; 269) |  |  |
| Trunk PT flexion 60º/s (Nm∙kg ^– 1^) | CG | 99.8 (90.8; 108.8) | 83.9 (74.9; 92.9) | 74.6 (65.2; 84.1) | 81.5 (71.2; 91.8) | 10.3 (-2.1; 22.6); p = 0.104; d = -0.65 | 1.0 (-11.9; 14.2); p = 0.875; d = -0.07 |
|  | > 50% | 100.9 (91.4; 110.4) | 80.7 (71.2; 90.2) | 84.9 (75.3; 94.6) | 82.5 (72.8; 92.3) |  |  |
| Trunk PT extension 120º/s (Nm∙kg ^– 1^) | CG | 163 (134; 193) | 163 (133; 192) | 150 (119; 180) | 182 (149; 216) | 42.7 (1.6; 83.9); p = **0.044**; d = -0.84 | 14.8 (-27.7; 57.3); p = 0.497; d = -0.29 |
|  | > 50% | 167 (137; 197) | 182 (152; 212) | 192 (160; 225) | 197 (166; 228) |  |  |
| Trunk PT flexion 120º/s (Nm∙kg ^– 1^) | CG | 67.8 (58.6; 77.0) | 58.3 (48.7; 67.8) | 49.3 (39.4; 59.2) | 56.8 (46.1; 67.5) | 7.7 (-4.7; 20.2); p = 0.224; d = -0.53 | 7.1 (-5.9; 20.0); p = 0.286; d = -0.48 |
|  | > 50% | 68.9 (59.4; 78.4) | 55.5 (45.8; 65.2) | 57.0 (46.9; 67.1) | 63.8 (54.1; 73.6) |  |  |
| Trunk extension total work 60º/s (J) | CG | 190 (171; 209) | 177 (158; 196) | 180 (159; 200) | 205 (183; 228) | 11.3 (-16.5; 39.1); p = 0.424; d = -0.30 | -7.8 (-37.0; 21.5); p = 0.604; d = 0.20 |
|  | > 50% | 185 (165; 205) | 188 (168; 208) | 191 (170; 212) | 198 (177; 219) |  |  |
| Trunk flexion total work 60º/s (J) | CG | 67.6 (60.9; 74.3) | 58.8 (52.1; 65.5) | 52.6 (45.6; 59.7) | 56.9 (49.3; 64.6) | 1.6 (-7.5; 10.7); p = 0.729; d = -0.74 | 4.2 (-13.7; 5.4); p = 0.392; d = -0.36 |
|  | > 50% | 68.0 (61.0; 74.9) | 55.7 (48.7; 62.6) | 54.2 (47.1; 61.3) | 61.1 (53.9; 68.3) |  |  |
| Trunk extension total work 120º/s (J) | CG | 109 (86.9; 132) | 105 (82.3; 127) | 102 (78.5; 126) | 124 (98.5; 150) | 28.8 (-2.8; 60.3); p = 0.076; d = -0.75 | 17.7 (-14.8; 50.3); p = 0.289; d = -0.46 |
|  | > 50% | 112 (88.4; 135) | 120 (96.9; 144) | 131 (105.8; 156) | 142 (117.7; 166) |  |  |
| Trunk flexion total work 120º/s (J) | CG | 42.0 (35.6; 48.5) | 33.4 (27.0; 39.9) | 27.8 (21.0; 34.6) | 32.3 (25.0; 39.6) | 6.0 (-2.7; 14.6); p = 0.704; d = -0.56 | 6-6 (-2.3; 15.6); p =0.147; d = -0.62 |
|  | > 50% | 41.9 (35.3; 48.5) | 30.6 (24.0; 37.2) | 33.8 (26.7; 40.8) | 38.9 (32.2; 45.7) |  |  |
| Time to PT extension 60º/s (Mseg) | CG | 455 (368; 542) | 456 (368; 543) | 384 (292; 476) | 291 (190; 393) | -60.3 (-186.4; 65.7); p = 0.349; d = 0.35 | 10.5 (-122.2; 143.2); p = 0.876; d = -0.06 |
|  | > 50% | 445 (353; 537) | 377 (285; 469) | 324 (229; 418) | 302 (207; 397) |  |  |
| Time to PT flexion 60º/s (Mseg) | CG | 465 (323; 607) | 626 (484; 768) | 560 (411; 710) | 598 (434; 762) | 79.1 (122.6; 280.8); p = 0.443; d = 0.30 | -66.2 (-278.9; 145.9); p = 0.542; d = 0.25 |
|  | > 50% | 474 (326; 622) | 518 (370; 667) | 481 (329; 634) | 532 (379; 684) |  |  |
| Time to PT extension 120º/s (Mseg) | CG | 240 (216; 264) | 239 (214; 264) | 219 (193; 244) | 227 (199; 255) | -31.3 (-66.7; 4.21); p = 0.09; d = 0.67 | -36.0 (18.7; -72.6); 0.06; d = 0.77 |
|  | > 50% | 219 (194; 245) | 209 (183; 234) | 188 (160; 215) | 191 (164; 217) |  |  |
| Time to PT flexion 120º/s (Mseg) | CG | 387 (339; 434) | 433 (383; 482) | 486 (435; 537) | 435 (379; 491) | -50.6 (-118.6; 17.5); p = 0.147; d = 0.62 | -0.4 (-71.2; 70.4); p = 0.992; d = 0.004 |
|  | > 50% | 376 (327; 425) | 468 (417; 518) | 436 (383; 489) | 435 (385, 485) |  |  |
| Note: Data are presented as estimated marginal mean (EMM) and 95% CI. Treatment effect was reported as estimated mean difference (EMD) and 95% CI. Statistical significance was considered when p < 0.05. Cohen’s d = (d).  Abbreviations: BS= bariatric surgery; CG= control group; >50%= exercise group, BW= body weight; LM= lean mass, PT= peak torque. | | | | | | | |

Supplementary table S3: Effects of a multicomponent exercise training attendance in absolute trunk muscle strength changes post-BS
