# Supplementary material for: Impact of a Multicomponent Exercise Training Program on Muscle Strength After Bariatric Surgery: A Randomized Controlled Trial
Source: Obes Surg. 2024 Mar 27;34(5):1704–16. doi: 10.1007/s11695-024-07173-w (PMC11031478; doi:10.1007/s11695-024-07173-w)
Supplement: Supplementary file 4 — (DOCX 23 kb) [file 11695_2024_7173_MOESM4_ESM.docx]

| Variable | Group | Pre-BS | 1-month post-BS | 6-months post-BS | 12-months post-BS | Treatment effect  Baseline vs 6-months | Treatment effect baseline vs 12-months |
| --- | --- | --- | --- | --- | --- | --- | --- |
| **Relative muscle strenght** |  |  |  |  |  |  |  |
| Knee PT extension 60°/s  Relative to BW (Nm∙kg ^– 1^) | CG | 1.32 (1.23; 1.40) | 1.34 (1.25; 1.42) | 1.49 (1.41; 1.58) | 1.68 (1.58; 1.77) | 0.2 (0.09; 0.32); **p = 0.001**; d = -1.36 | 0.1 (-0.03; 0.23); p = 0.133; d = -0.65 |
|  | >50% | 1.34 (1.24; 1.43) | 1.39 (1.29; 1.48) | 1.70 (1.61; 1.79) | 1.77 (1.68; 1.87) |  |  |
| Knee PT flexion 60°/s  Relative to BW(Nm∙kg ^– 1^) | CG | 0.69 (0.64; 0.75) | 0.67 (0.61; 0.73) | 0.81 (0.76; 0.87) | 0.92 (0.85; 0.98) | 0.1 (0.02; 0.18); p = **0.012**; d = -0.98 | 0.1 (0.03; 0.20); p = **0.009;** d = -1.11 |
|  | >50% | 0.69 (0.63; 0.75) | 0.72 (0.65; 0.78) | 0.92; (0.85; 0.98) | 1.03 (0.96; 1.09) |  |  |
| Knee PT extension 60°/s  Relative to LM (Nm∙kg ^– 1^) | CG | 2.73 (2.59; 2.88) | 2.80 (2.66; 2.94) | 2.70 (2.56; 2.85) | 2.79 (2.63; 2.96) | 0.2 (0.04; 0.43); **p = 0.017**; d = -0.91 | -0.05 (-0.27; 0.16); p = 0.638; d = 0.20 |
|  | >50% | 2.77 (2.62; 2.92) | 2.90 (2.75; 3.05) | 2.94 (2.79; 3.09) | 2.74 (2.58; 2.90) |  |  |
| Knee PT flexion 60°/s  Relative to LM (Nm∙kg ^– 1^) | CG | 1.40 (1.31; 1.50) | 1.39 (1.30; 1.49) | 1.47 (1.37; 1.57) | 1.50 (1.39; 1.61) | 0.09 (-0.04; 0.22); p = 0.158; d = -0.54 | 0.08 (-0.06; 0.23); p = 0.261; d = -0.47 |
|  | >50% | 1.40 (1.30; 1.50) | 1.46 (1.36; 1.56) | 1.56 (1.46; 1.67) | 1.58 (1.48; 1.69) |  |  |
| Knee PT extension 60°/s  Relative to thigh LM (Nm∙kg ^– 1^) | CG | 21.7 (20.5; 22.9) | 19.9 (18.7; 21.1) | 18.1 (16.9; 19.3) | 18.3 (17.0; 19.6) | 2.4 (0.77; 3.94); p **= 0.004**; d = -1.14 | 0.5 (-1.21; 2.28); p = 0.546; d = -0.26 |
|  | >50% | 22.2 (21.0; 23.5) | 20.8 (19.6; 22.1) | 20.4 (19.2; 21.7) | 18.8 (17.5; 20.2) |  |  |
| Knee PT flexion 60°/s  Relative to thigh LM (Nm∙kg ^– 1^) | CG | 11.3 (10.4; 12.1) | 11.5 (10.6; 12.3) | 12.8 (11.9; 13.7) | 13.3 (12.3; 14.3) | 0.7 (-0.44; 1.88); p = 0.225; d = -0.49 | 0.7 (-0.54; 2.00); p = 0.262; d = -0.05 |
|  | >50% | 11.3 (10.4; 12.2) | 12.0 (11.1, 12.9) | 13.6 (12.6; 14.5) | 14.0 (13.1; 15.0) |  |  |
| Knee PT extension 180°/s  Relative to BW (Nm∙kg ^– 1^) | CG | 0.88 (0.82; 0.94) | 0.87 (0.81; 0.93) | 0.98 (0.92; 1.04) | 1.16 (1.09; 1.23) | 0.1 (0.03; 0.19); **p = 0.006**; d = -1.09 | 0.007 (-0.08; 0.09); p = 0.877; d = -0.07 |
|  | >50% | 0.90 (0.84; 0.97) | 0.90 (0.84; 0.97) | 1.09 (1.03; 1.15) | 1.17 (1.10; 1.23) |  |  |
| Knee PT flexion 180°/s  Relative to BW (Nm∙kg ^– 1^) | CG | 0.50 (0.45; 0.55) | 0.50 (0.45; 0.55) | 0.61 (0.56; 0.66) | 0.69 (0.64; 0.75) | 0.05 (-0.02; 0.11) p = 0.161; d = -0.54 | 0.04 (-0.03; 011); p = 0.299; d = -0.44 |
|  | >50% | 0.50 (0.45; 0.55) | 0.49 (0.44; 0.54) | 0.65 (0.60; 0.70) | 0.73 (0.68; 0.79) |  |  |
| Knee PT extension 180°/s  Relative to LM (Nm∙kg ^– 1^) | CG | 1.84 (1.74; 1.93) | 1.82 (1.73; 1.92) | 1.79 (1.70; 1.89) | 1.89 (1.79; 2.00) | 0.1 (-0.02; 0.24); p = 0.093; d = -0.65 | -0.04 (-0.18; 0.10); p = 0.578; d = 0.23 |
|  | >50% | 1.88 (1.78; 1.98) | 1.88 (1.78; 1.98) | 1.90 (1.80; 2.01) | 1.85 (1.75; 1.96) |  |  |
| Knee PT flexion 180°/s  Relative to LM (Nm∙kg ^– 1^) | CG | 1.02 (0.95; 1.10) | 1.02 (0.95; 1.10) | 1.07 (1.00; 1.15) | 1.13 (1.04; 1.22) | 0.05 (-0.06; 0.15); p = 0.356; d = -0.36 | -0.01 (-0.12; 0.11); 0.915; d = 0.05 |
|  | >50% | 1.02 (0.94; 1.10) | 1.04 (0.95; 1.12) | 1.12 (1.04; 1.20) | 1.12 (1.04; 1.21) |  |  |
| Knee PT extension 180°/s  Relative to thigh LM (Nm∙kg ^– 1^) | CG | 14.8 (13.9; 15.7) | 15.0 (14.1; 16.0) | 15.7 (14.8; 16.6) | 16.9 (15.9; 18.0) | 0.82 (-0.41; 2.06); p = 0.194; d = -0.53 | -0.58 (-1.93; 0.77); p = 0.402; d = 0.37 |
|  | >50% | 15.2 (14.2; 16.2) | 15.4 (14.4; 16.4) | 16.5 (15.6; 17.5) | 16.4 (15.3; 17.4) |  |  |
| Knee PT flexion 180°/s  Relative to thigh LM (Nm∙kg ^– 1^) | CG | 8.20 (7.46; 8.95) | 8.49 (7.74; 9.23) | 9.40 (8.65; 10.1) | 10.2 (9.35; 11.0) | 0.36 (-0.61; 1.34); p = 0.468; d = -0.29 | -0.12(-1.19; 0.95); p = 0.825; d = 0.10 |
|  | >50% | 8.30 (7.53; 9.08) | 8.45 (7.67; 9.22) | 9.76 (8.98; 10.5) | 10.1 (9.24; 10.9) |  |  |
| Note: Data are presented as estimated marginal mean (EMM) and 95% CI. Treatment effect was reported as estimated mean difference (EMD) and 95% CI. Statistical significance was considered when p < 0.05. Cohen’s d = (d).  Abbreviations: BS= bariatric surgery; CG= control group; >50%= exercise group, BW= body weight; LM= lean mass, PT= peak torque. | | | | | | | |

Supplementary table S4: Effects of a multicomponent exercise training attendance in relative knee muscle strength changes post-BS
