# Supplementary material for: Impact of a Multicomponent Exercise Training Program on Muscle Strength After Bariatric Surgery: A Randomized Controlled Trial
Source: Obes Surg. 2024 Mar 27;34(5):1704–16. doi: 10.1007/s11695-024-07173-w (PMC11031478; doi:10.1007/s11695-024-07173-w)
Supplement: Supplementary file 5 — (DOCX 23 kb) [file 11695_2024_7173_MOESM5_ESM.docx]

Supplementary table S5: Effects of a multicomponent exercise training attendance in relative trunk muscle strength changes post-BS

| Variable | Group | Pre-BS | 1-month after-BS | 6-months after-BS | 12-months after-BS | Treatment effect  Baseline vs 6-months | Treatment effect baseline vs 12-months |
| --- | --- | --- | --- | --- | --- | --- | --- |
| **Relative trunk muscle strength** |  |  |  |  |  |  |  |
| Trunk PT extension 60º/s  Relative to BW (Nm∙kg ^– 1^) | CG | 2.17 (1.91; 2.43) | 2.10 (1.84; 2.36) | 2.74 (2.46; 3.02) | 3.55 (3.24; 3.87) | 0.50 (0.12; 0.88); p = **0.010**; d = -0.98 | 0.27 (-0.68; 0.13); p = 0.187; d = 0.54 |
|  | >50% | 2.13 (1.92; 2.42) | 2.43 (2.16; 2.71) | 3.24 (2.96; 3.52) | 3.28 (3.00; 3.56) |  |  |
| Trunk PT extension 120º  Relative to BW (Nm∙kg ^– 1^) | CG | 0.61 (0.51; 0.72) | 0.59 (0.48; 0.69) | 0.60 (0.49; 0.71) | 0.79 (0.66; 0.91) | 0.11 (-0.03; 0.25); p = 0.134; d = -0.67 | 0.06 (-0.09; 0.21); p = 0.425; d = -0.38 |
|  | >50% | 0.62 (0.51; 0.73) | 0.55 (0.44; 0.66) | 0.71 (0.59; 0.82) | 0.85 (0.74; 0.96) |  |  |
| Trunk PT flexion 60º/s  Relative to BW (Nm∙kg ^– 1^) | CG | 0.88 (0.79; 0.98) | 0.83 (0.73; 0.92) | 0.91 (0.81; 1.01) | 1.06 (0.95; 1.71) | 0.14 (0.00; 0.27); p = **0.047**; d = -0.82 | 0.06 (-0.08; 0.20); p = 0.392; d = -0.38 |
|  | >50% | 0.90 (0.80; 1.00) | 0.82 (0.72; 0.92) | 1.04 (0.94; 1.14) | 1.12 (1.02; 1.23) |  |  |
| Trunk PT flexion 120º  Relative to BW (Nm∙kg ^– 1^) | CG | 0.61 (0.51; 0.72) | 0.59 (0.48; 0.69) | 0.60 (0.49; 0.71) | 0.79 (0.66; 0.91) | 0.11 (-0.03; 0.25); p = 0.134; d = 0.67 | 0.06 (-0.09; 0.21); p = 0.425; d = -0.38 |
|  | >50% | 0.62 (0.51; 0.73) | 0.55 (0.44; 0.66) | 0.71 (0.59; 0.82) | 0.85 (0.74; 0.96) |  |  |
| Trunk PT extension 60º/s  Relative to total LM (Nm∙kg ^– 1^) | CG | 4.42 (3.93; 4.91) | 4.27 (3.78; 4.77) | 4.97 (4.45; 5.49) | 5.91 (5.31; 6.51) | 0.67 (-0.04; 1.39); p = 0.067; d = -0.68 | -0.67 (-1.44; 0.10); p = 0.091; d = 0.68 |
|  | >50% | 4.30 (3.78; 4.82) | 4.92 (4.40; 5.44) | 5.64 (5.11; 6.18) | 5.24 (4.71; 5.78) |  |  |
| Trunk PT extension 120º  Relative to total LM (Nm∙kg ^– 1^) | CG | 2.35 (2.00; 2.70) | 2.25 (1.88; 2.62) | 2.04 (1.66; 2.42) | 2.37 (1.94; 2.79) | 0.3 (-01.9; 0.82); p = 0.220; d = -0.54 | 0.2 (-0.31; 0.75); p = 0.417; d = -0.38 |
|  | >50% | 2.42 (2.05; 2.80) | 2.19 (1.81; 2.57) | 2.36 (1.96; 2.75) | 2.59 (2.21; 2.97) |  |  |
| Trunk PT flexion 60º/s  Relative to total LM (Nm∙kg ^– 1^) | CG | 1.77 (1.60; 1.93) | 1.65 (1.48; 1.82) | 1.60 (1.42; 1.77) | 1.72 (1.52; 1.92) | 0.17 (-0.07; 0.42); p = 0.159; d = -0.57 | 0.01 (-0.25; 0.27); p = 0.948; d = -0.03 |
|  | >50% | 1.84 (1.67; 2.02) | 1.62 (1.45; 1.80) | 1.77 (1.59; 1.95) | 1.73 (1.55; 1.92) |  |  |
| Trunk PT flexion 120º  Relative to total LM (Nm∙kg ^– 1^) | CG | 1.18 (0.09; 1.36) | 1.12 (0.94; 1.31) | 1.04 (0.84; 1.23) | 1.21 (0.99; 1.43) | 0.14 (-0.11; 0.40) p = 0.267; d = -0.50 | 0.11 (-0.16; 0.37); p = 0.438; d = -0.37 |
|  | >50% | 1.22 (1.03; 1.41) | 1.09 (0.90; 1.29) | 1.18 (0.98; 1.38) | 1.32 (1.13; 1.51) |  |  |
| Trunk PT extension 60º/s  Relative to trunk LM (Nm∙kg ^– 1^) | CG | 8.75 (7.76; 9.74) | 8.61 (7.62; 9.60) | 9.89 (8.85; 10.9) | 11.7 (10.5; 12.9) | 1.29 (-0.14; 2.72); p = 0.080; d = -0.65 | -1.40 (-2.94; 0.14); p = 0.077; d = 0.71 |
|  | >50% | 8.49 (7.45; 9.54) | 9.83 (8.78; 10.9) | 11.2 (10.1; 12.3) | 10.3 (9.20; 11.4) |  |  |
| Trunk PT extension 120º  Relative to trunk LM (Nm∙kg ^– 1^) | CG | 5.54 (4.33; 6.76) | 6.16 (4.92; 7.40) | 6.21 (4.94; 7.48) | 7.85 (6.43; 9.28) | 1.93 (0.22; 3.65); p = **0.029**; d = -0.94 | 0.26 (-1.54; 2.06); p = 0.778; d = -0.13 |
|  | >50% | 5.79 (4.52; 7.05) | 7.36 (6.10; 8.62) | 8.14 (6.80; 9.49) | 8.11 (6.82; 9.41) |  |  |
| Trunk PT flexion 60º/s  Relative to trunk LM (Nm∙kg ^– 1^) | CG | 3.54 (3.21; 3.88) | 3.32 (2.99; 3.65) | 3.16 (2.81; 3.51) | 3.42 (3.03; 3.82) | 0.37 (-0.10; 0.84); p = 0.127; d = -0.61 | 0.001 (-0.51; 0.50); p = 0.996; d = 0.002 |
|  | >50% | 3.64 (3.29; 3.99) | 3.27 (2.92; 3.62) | 3.53 (3.17; 3.89) | 3.42 (3.06; 3.78) |  |  |
| Trunk PT flexion 120º  Relative to trunk LM (Nm∙kg ^– 1^) | CG | 2.38 (2.02; 2.75) | 2.29 (1.91; 2.67) | 2.08 (1.68; 2.47) | 2.41 (1.98; 2.85) | 0.31 (-0.20; 0.81); p = 0.240; d = -0.52 | 0.20 (-0.34; 0.73); p = 0.479; d = -0.33 |
|  | >50% | 2.45 (2.07; 2.83) | 2.21 (1.82, 2.60) | 2.38 (1.98; 2.78) | 2.61 (2.22; 3.00) |  |  |
| Note: Data are presented as estimated marginal mean (EMM) and 95%CI. Treatment effect was reported as estimated mean difference (EMD) and 95%CI. Statistical significance was considered when p < 0.05, and Cohen’s d = (d).  Abbreviations: BS= bariatric surgery; CG= control group; >50%= exercise group, BW= body weight; LM= lean mass, PT= peak torque | | | | | | | |
